# Automatic therapy planning based on concept drift

**DOI:** 10.3389/fresc.2026.1874544

**Published:** 2026-08-05

**Authors:** Miranda Ramírez-Cruz, Luis Enrique Sucar, Eduardo F. Morales, Jesús Joel Rivas

**Affiliations:** 1 Department of Computer Science, Instituto Nacional de Astrofísica, Óptica y Electrónica, Santa María Tonantzintla, Puebla, México; 2 Department of Computer Science, Universidad Tecnológica de Tecamachalco, Tecamachalco, Puebla, México

**Keywords:** concept drift, Markov decision process, motor rehabilitation, therapy planning, virtual therapy

## Abstract

Static rehabilitation protocols often struggle to keep pace with the dynamic, non-linear reality of human motor recovery. Traditional therapy models frequently assume stationarity, relying on fixed performance schemes that fail to capture the highly individualized nature of human recovery and skill acquisition. This paper proposes an adaptive decision-support framework that integrates concept drift detection within Bayesian Networks to drive a Markov Decision Process (MDP) for automated therapy planning. By continuously monitoring real-time data streams, the system can identify both parametric and structural shifts in a patient’s motor control during virtual rehabilitation sessions. These detected drifts serve as signal for the MDP, which searches and recommends an therapeutic adjustment. As a proof of concept, the framework was evaluated using data from healthy participants interacting with the platform and assessed through expert clinical feedback. In this preliminary evaluation, the generated policies balanced engagement with progression, rewarding improvement while penalizing task overload and frustration. While validation in clinical populations remains as future work, the integration of probabilistic tracking and sequential decision-making offers a promising basis for personalized, data-driven virtual rehabilitation in which therapeutic adjustments can evolve alongside the user.

## 1 Introduction

Cerebrovascular accidents (CVAs), commonly known as strokes, are a leading cause of long-term disability and represent a major global healthcare challenge (1). Among stroke survivors, an estimated 33% to 78% experience motor impairments, particularly affecting the upper extremities (2). These impairments can range from diminished fine motor control to severe limitations, including the complete loss of arm mobility. In response to this widespread issue, innovative rehabilitation strategies are continually being developed to support functional recovery (1).

Virtual and computer-assisted therapies have shown promising results, offering engaging and scalable approaches for individuals with motor deficits and upper limb limitations following a stroke. Gesture Therapy (GT) is a computer vision-based rehabilitation system that enables individuals to perform arm movement exercises either at home or in rehabilitation clinics, with periodic supervision from a therapist (3, 4).

Rehabilitation is a long and demanding process that relies heavily on sustained patient engagement and adherence. However, maintaining motivation throughout this journey can be challenging, particularly when progress is slow or difficult to perceive. Furthermore, traditional therapy models often assume stationarity, relying on static performance schemes that fail to capture the evolving, highly individualized nature of human recovery. An adaptive planning system has the potential to empower patients by offering clear, personalized therapeutic plans, real-time progress tracking, and continuous feedback. These elements can foster a greater sense of control and involvement, enhancing patient motivation and commitment to the rehabilitation program, key aspects for obtaining positive recovery outcomes.

Moreover, such a system can serve as a valuable support tool for clinicians (5). By delivering data-driven, evidence-based recommendations, it enables healthcare centers to optimize the use of therapists’ time and expertise, allowing them to focus on direct patient care and complex clinical decisions. Consequently, the system not only enhances the efficiency of rehabilitation workflows but also contributes to the overall quality and accessibility of rehabilitation services.

Several approaches to adaptive rehabilitation have been proposed. Adaptive and personalized rehabilitation systems aim to tailor therapy to a patient’s evolving abilities rather than following a fixed protocol. In virtual rehabilitation, most platforms adjust the difficulty of serious games through optimization rules or conditional statements driven by in-game performance (6). Reinforcement learning has been explored as a more flexible alternative, since it requires no pretraining and can adapt a game in real time from observed outcomes (7). Sekhavat and Zarei (8) framed difficulty adjustment as a multiple-objective problem and proposed Multiple-Periodic Reinforcement Learning to balance challenge against user satisfaction, and Pelosi et al. (6) used a Q-learning approach that adapted a reaching task to a patient’s kinematic performance. Hocine et al. (9) combined a Markov decision process with a therapist-guided reinforcement learning algorithm to adapt game difficulty for motor rehabilitation. These systems adapt difficulty within a single game, whereas the present work decides among game categories along a clinically defined hierarchy and incorporates kinematic trends and self-reported metrics rather than success and failure alone.

To bridge the gap between static rehabilitation protocols and dynamic patient needs, this paper proposes an adaptive decision-support framework that integrates concept drift detection with a Markov Decision Process (MDP). By modeling patient performance through Bayesian Networks, the system continuously monitors data streams for drift, which are significant shifts in the underlying data distribution that indicate changes in motor control, such as skill acquisition or fatigue. Concept drift detection serves as signal for MDP to dynamically adjust the therapy plan, ensuring the selected exercises consistently align with the patient’s current capabilities. This approach establishes a personalized, non-stationary model that reliably adapts to the complex nature of motor recovery.

To validate the drift detection system, experiments were conducted across synthetic, semi-synthetic, and real-world datasets derived from healthy participants interacting with the Gesture Therapy platform, as an initial proof-of-concept validation prior to deployment with clinical populations. The results demonstrate that the method effectively identifies and adapts to both parametric and structural shifts in the data distribution, maintaining model stability and interpretability under evolving conditions. Furthermore, integrating this drift-aware model with the proposed MDP framework successfully generated adaptive therapy recommendations that aligned with patient progress and established therapeutic procedures. Ultimately, the primary contributions of this work are the application of a concept drift adaptation mechanism to dynamically update Bayesian Network models during motor recovery, and the development of a decision-making model that translates these adapted states into personalized therapy plans.

This paper explores the design, implementation, and validation of this adaptive framework, detailing its continuous loop from initial performance tracking to final recommendation. First, the paper outlines how Bayesian Networks translate patient kinematics into detectable, critical shifts in motor performance, and details the mechanism used for detecting these changes. Building on this detection framework, the decision-making process is formalized as a Markov Decision Process (MDP), which evaluates the participant’s evolving clinical state to recommend targeted adjustments. Finally, the practical viability and alignment of the integrated system are demonstrated through testing with real-world data from the Gesture Therapy platform, supported by qualitative validation from clinical experts.

We emphasize that this work is presented as a proof of concept. The framework is evaluated with healthy participants to establish baseline feasibility and to map the natural trajectory of skill acquisition, with validation in stroke survivors identified as the essential next step.

## 2 Background

Before an adaptive system can decide how to adapt a therapy plan, it must first recognize when a change is necessary. This requires a system to capture patient performance and a reliable way to interpret it.

### 2.1 Clinical environment: gesture therapy

**Figure 1:**
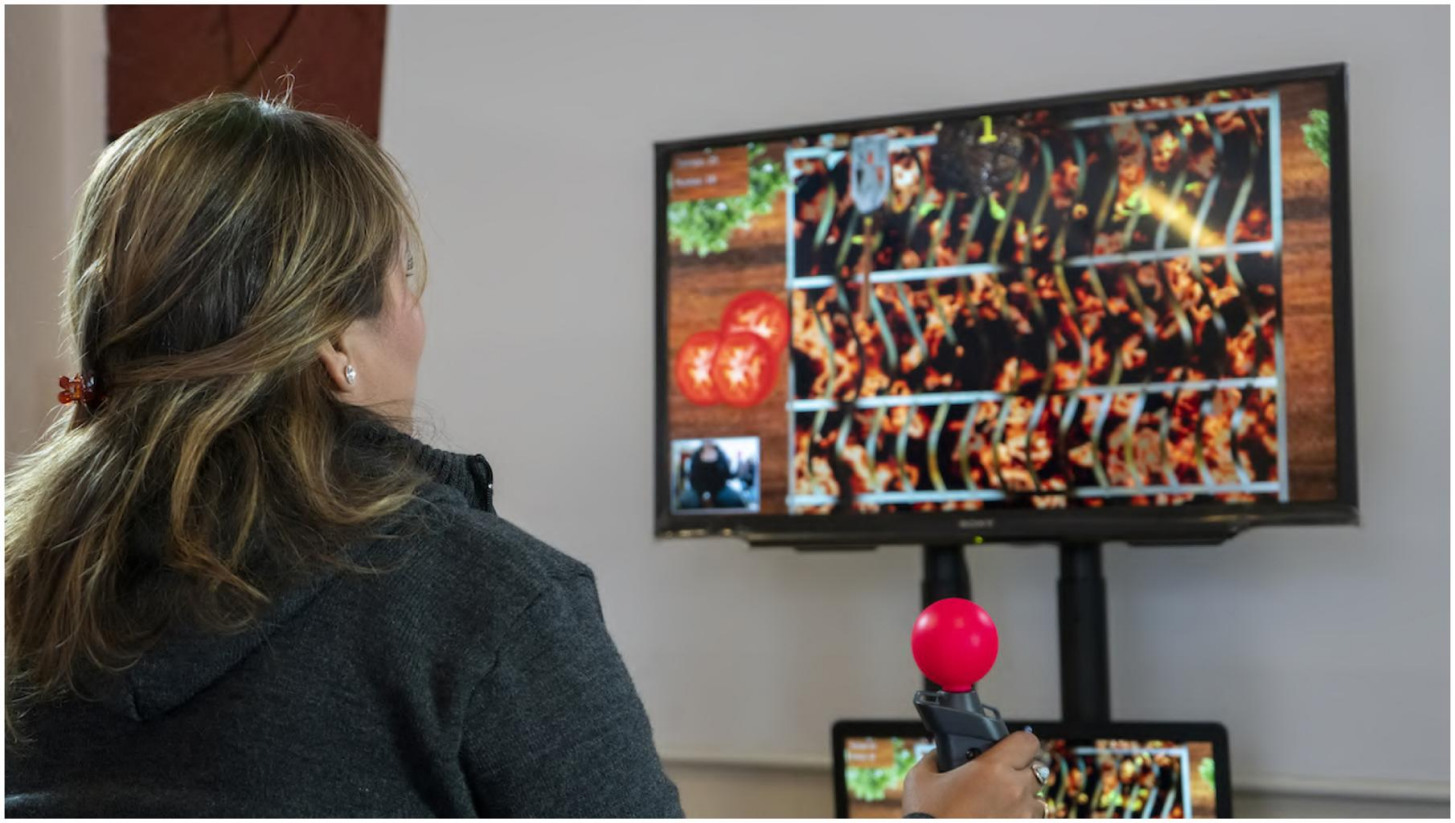
Gesture therapy system: a participant uses the gripper controller to play one of the gesture therapy games (Grill) while the system tracks their movement.

Gesture Therapy (GT) serves as the primary interactive environment for this framework (3, 4). GT is a virtual reality-based platform designed to assist stroke survivors in recovering upper extremity mobility. The system comprises a simulated environment of “serious games,” a computer vision tracking system, and a handgrip controller equipped with a pressure sensor. As patients interact with the games, GT continuously captures different streams of kinematic and performance data, including spatial coordinates, velocity, acceleration, path tortuosity, and grip pressure (Figure 1).

Since the platform is built on rehabilitation principles such as task-oriented training and repetition, a patient’s interaction with the system naturally evolves. As they engage with the games over multiple sessions, they exhibit learning curves, experience fatigue, or may even regain motor function. Consequently, the data generated by GT is inherently non-stationary.

### 2.2 Gesture therapy games

Gesture Therapy offers a set of “serious games”, each casting an everyday scenario as a motor task. The games differ in the movements they require, from broad reaching to controlled rotation to sustained grip, and in the constraints placed on the player, such as obstacles, target speed, and time limits. A representative image of each is shown in Figure 3.

- **Kitchen:** The player reaches toward targets that appear one at a time within a narrow area, with no obstacles and long intervals between appearances that allow ample reaction time.
- **Grill:** Targets appear in faster succession and for shorter durations, so the player must reach under time pressure. No obstacles are present.
- **Library:** Obstacles are introduced, the target area is broadened, and targets move more quickly, requiring the player to plan reaches around the environment.
- **Garage:** A rotational task in which the player follows a more complex trajectory, succeeding through controlled rotation of the arm and wrist rather than simple reaching.
- **Stable:** Combines a simple vertical arm movement with maintaining and then releasing finger pressure on the gripper, introducing grip modulation alongside arm motion.
- **Pond:** Requires displacements across the entire workspace toward targets that appear randomly and move unsteadily, combining broad compound arm movements with pressure control.
- **Archery:** The player maintains steady pressure and arm stability to maximize points, while a countdown timer adds time pressure, demanding fine grip control under load.

### 2.3 Game hierarchy

**Figure 2:**
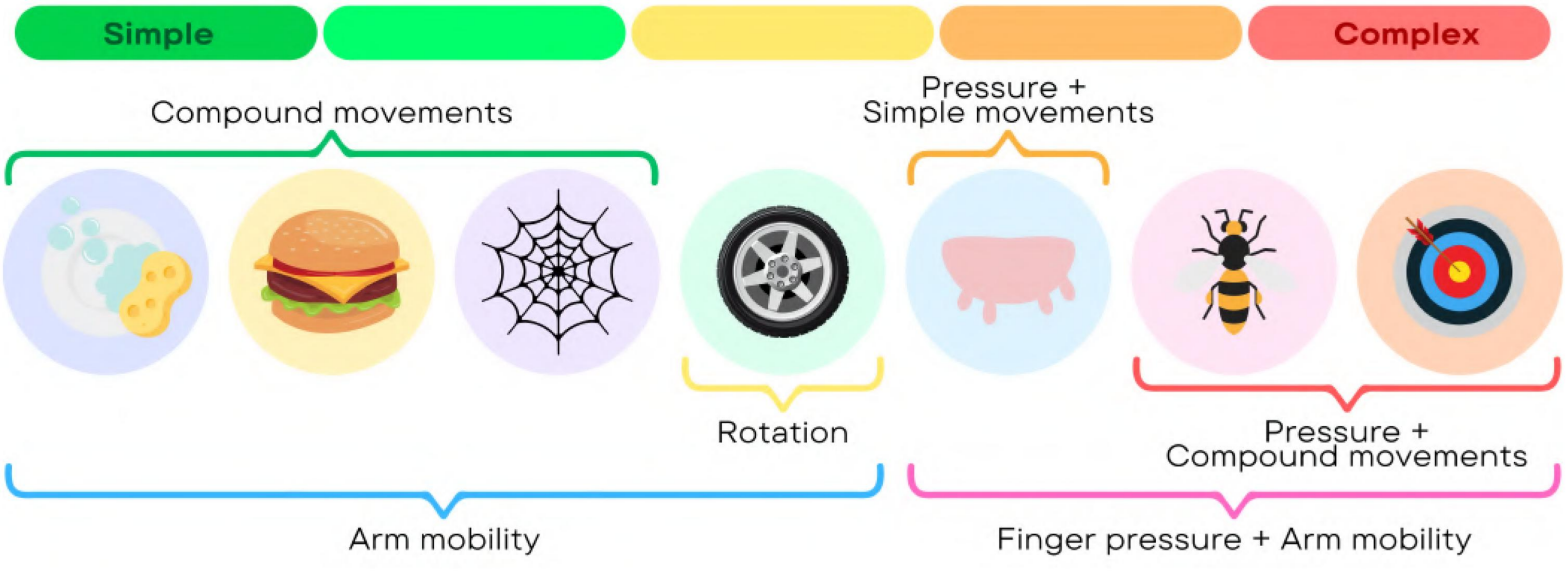
Game difficulty hierarchy aligned with Fugl-Meyer recovery path.

To ensure the framework’s automated decisions remain therapeutically valid, the available actions are constrained by a hierarchy that arranges these games along the natural progression of neurological rehabilitation, scaling from gross proximal movements to fine distal control. The ordering is modeled after the Fugl-Meyer assessment of motor function recovery (17) and is summarized in Figure 2:

- **Compound Movements (Arm Mobility):** General arm control through shoulder and elbow movements toward spatial targets. Within this category, difficulty rises from Kitchen (slow, narrow, no obstacles) to Grill (added time pressure) to Library (obstacles, wider area, faster targets).
- **Rotation:** Builds on basic shoulder and elbow mobility by engaging the more complex joint control of arm and wrist rotation, represented by Garage.
- **Finger Pressure & Simple Movements:** Shifts the focus toward hand and finger control by pairing simple arm trajectories with grip-force modulation, represented by Stable.
- **Pressure & Compound Movements:** The most advanced category, requiring coordinated use of shoulder, elbow, wrist, and fingers across the full workspace under time constraints. Pond and Archery integrate broad arm trajectories with continuous grip modulation, demanding both gross and fine motor control.

**Figure 3:**
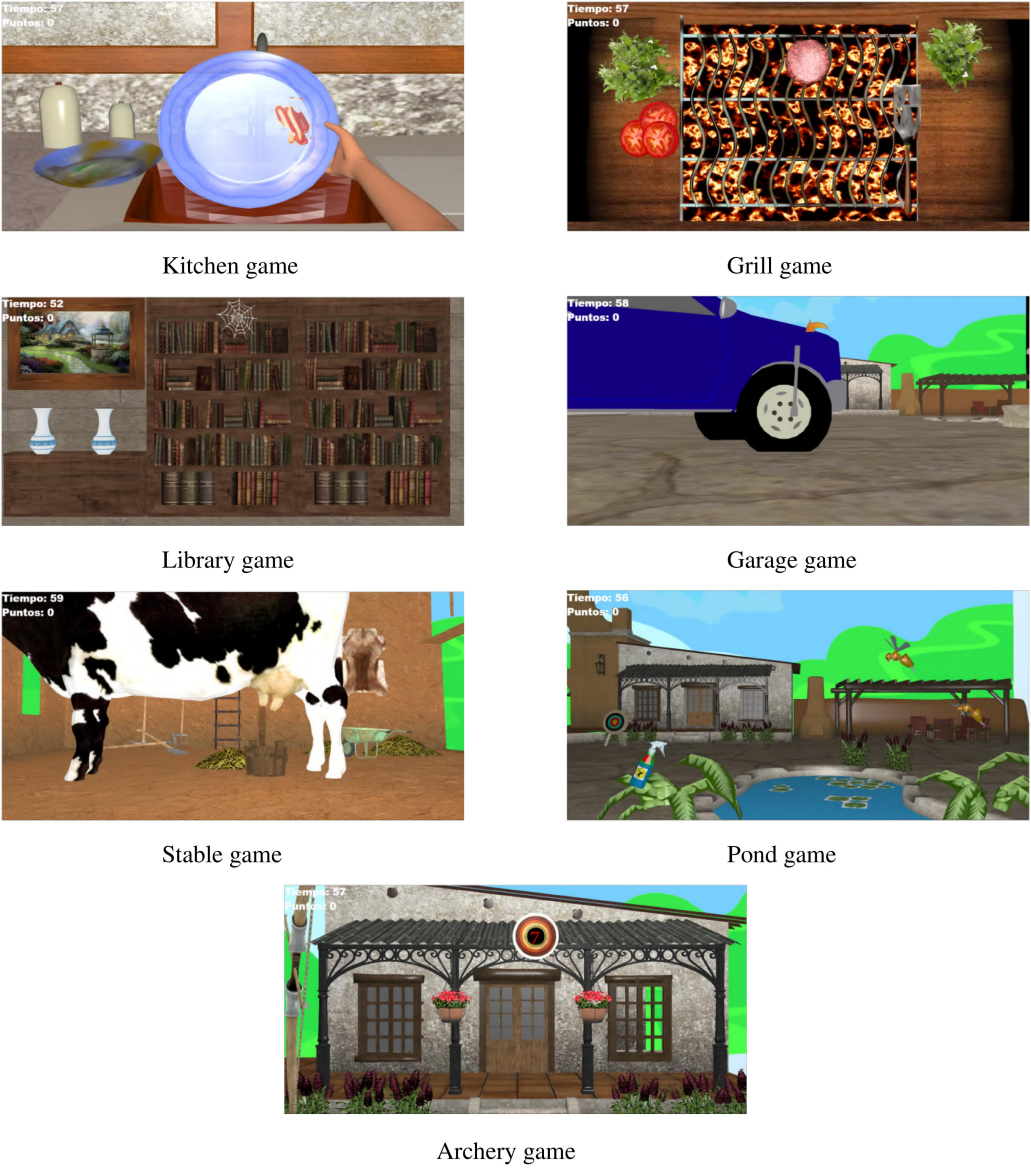
Gesture therapy games.

### 2.4 Modeling patient evolution with Bayesian networks

To make sense of the continuous data stream, Bayesian Networks (BNs) are used to model the probabilistic dependencies among a patient’s gaming metrics and movement parameters (10). A BN provides a highly interpretable framework, explicitly mapping out how variables influence one another during a specific phase of recovery. Variables set to describe the user’s performance are listed in Table 1.

**Table 1:** Variables used for patient performance modeling as a BN.

Variable	Description
Velocity (V)	Speed of the user’s movement with the handgrip controller.
Acceleration (A)	Rate of change of the movement velocity.
Displacement (Dx,Dy)	Magnitude of movement along the individual spatial axes.
Vector Disp. (Vd)	2D movement magnitude between consecutive spatial positions.
Tortuosity (T)	Measure of path deviation from a straight line (11).
Motion Range (Rs)	Difference between the maximum and minimum displacement magnitudes.

However, if a predictive model is trained exclusively on a patient’s initial baseline data, its performance may degrade as the patient improves or alters their behavior. To prevent the therapy planning from relying on outdated assumptions, the system must recognize when the underlying data distribution has shifted.

### 2.5 Concept drift detection

To recognize when a patient’s underlying performance distribution has shifted, we employ a drift detection mechanism based on probabilistic inference over temporal sliding windows (12). Specifically, as new streaming data arrives, a subset of variables from a given sample is randomly selected as observed evidence. The states of the remaining unobserved variables are then estimated via forward logic sampling using the current Bayesian Network (13). By comparing these inferred states against the actual true values of the samples, the system calculates a coincidence percentage (14).

This inference procedure is continuously evaluated across sliding windows to track the model’s predictive reliability over time. A concept drift event is formally flagged when the average coincidence percentage drops below a predetermined threshold over consecutive windows, signaling that the current model assumptions are outdated and no longer accurately represent the patient’s data.

In practice, inference reliability is tracked over a sliding window of 50 samples advanced in steps of 12, with two variables drawn at random as observed evidence at each step. A drift event is flagged when the mean coincidence percentage falls below 80 percent across consecutive windows. To avoid reacting to short-term fluctuations, while no drift is detected the samples from successive windows are aggregated, so detection draws on a broader history rather than a single noisy window. These values were set empirically to balance responsiveness against stability, and the detector’s robustness to them was assessed across 100 synthetic experiments spanning perturbation magnitudes Cp in [0.05, 0.35], sample counts in [5,000, 15,000], and 2 to 6 segments.

Once a drift is flagged, the framework distinguishes parametric from structural drift before adapting. It relearns the network structure on the recent window using a score-based search and compares it with the reference structure, the one in place when drift was detected, using the Structural Hamming Distance. If the distance is below a set threshold the change is treated as parametric and only the conditional probability tables are updated; if it is above, the change is treated as structural and the topology is relearned and its parameters reestimated. This decision rule is what separates, in practice, a patient refining an existing strategy from a patient adopting a new movement strategy:

- **Parametric Drift (Changes in Conditional Probabilities):** In a BN, parametric drift occurs when the probability distributions shift, but the underlying network structure remains stable. Clinically, this can translate to a patient refining an existing skill. The patient is utilizing the same biomechanical strategy to perform the movement, but their execution has evolved. For example, they may be getting faster at a known task, gradually increasing their range of motion, or exhibiting smoother trajectories with fewer hesitations. The relationships between their motor variables remain intact, but the magnitude and efficiency of the movements improves (or degrades due to fatigue).
- **Structural Drift (Changes in Network Topology):** Structural drift occurs when the actual dependencies (edges) between variables in the network change. Clinically, this could mean that the patient has fundamentally altered how they perform the movement. For instance, an early-stage patient might rely heavily on momentum, where raw velocity directly dictates their spatial displacement. However, as they recover fine-motor control, these variables may decouple as movements become more pre-planned and goal-directed rather than reactive. Alternatively, structural drift could indicate that a patient has developed a new compensatory movement strategy to overcome a difficult task, completely changing the biomechanical dependencies of their arm and hand.

By distinguishing between a patient improving in an specific area (parametric drift) and a patient adopting a completely new movement strategy (structural drift), the system gains a better understanding of the recovery process. This detected drift acts as signal for the Markov Decision Process (MDP), prompting the system to adjust the therapy plan to meet the patient’s new clinical state by the end of the session.

## 3 Adaptive therapy planning

**Figure 4:**
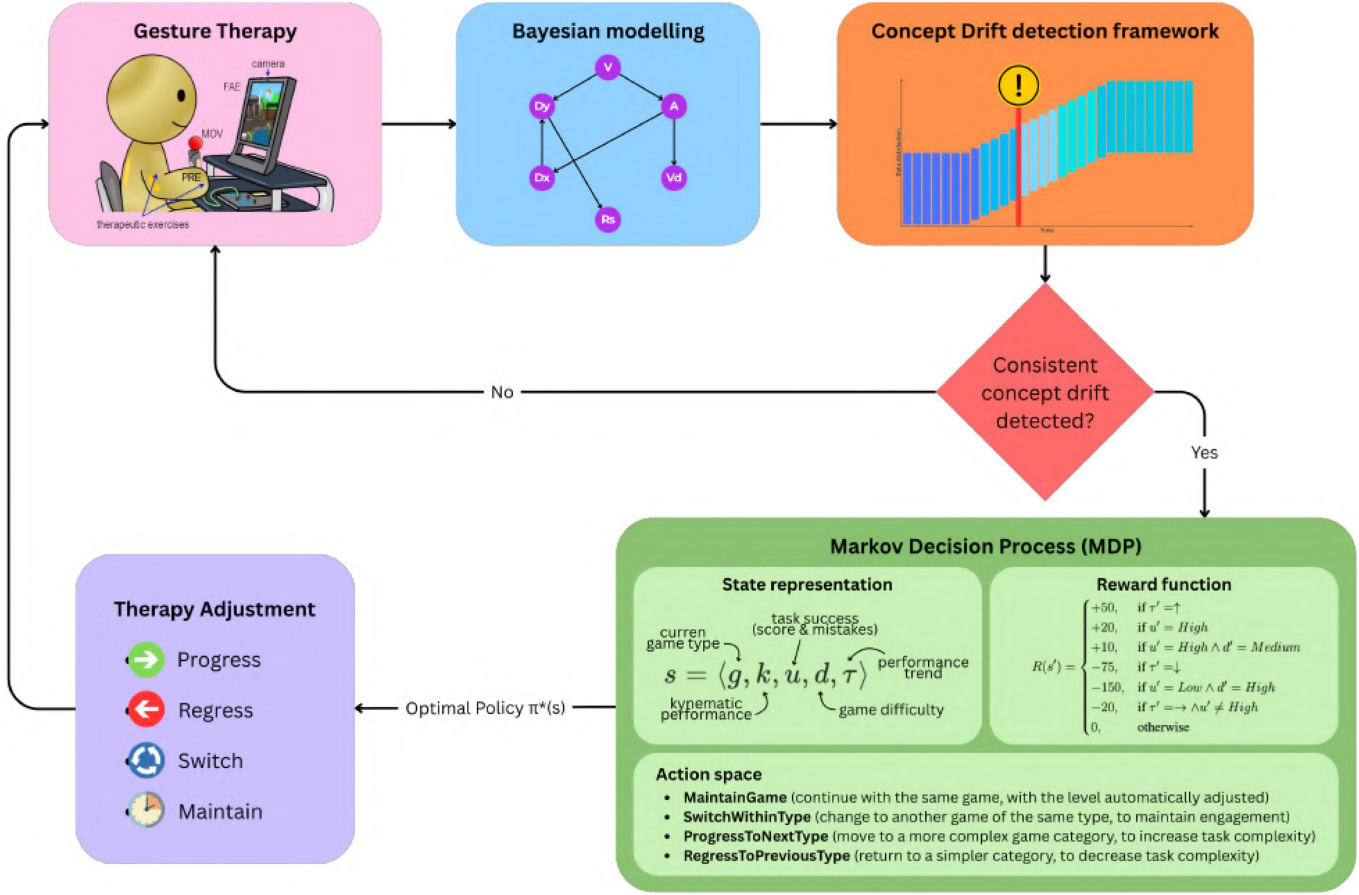
Adaptation framework general diagram.

Once concept drift presence is confirmed, signaling a significant change in the patient’s motor control, the system must determine the most appropriate clinical strategy. Rather than relying on simple if-then rules, the therapy planning process is formalized as a Markov Decision Process (MDP) (Figure 4). This framework allows the system to model sequential decision-making under uncertainty, effectively evaluating the patient’s evolving state to recommend the optimal subsequent exercise (10).

A Markov decision process is a natural formalism for this setting, since it models sequential decision making when outcomes are uncertain, representing the patient’s condition as a state, the therapeutic options as actions, the uncertain response to an action as a transition distribution, and the clinical value of an outcome as a reward (15). Markov decision processes have been used to support sequential clinical treatment decisions under uncertainty, for example the timing of living-donor liver transplantation (15). Compared with simple rule-based adaptation, which applies fixed if-then thresholds and does not weigh the downstream consequences of a decision, an MDP selects actions by their expected long-term therapeutic value, which is what allows the planner to model and decide under the uncertainty inherent in motor recovery.

### 3.1 MDP therapy modelling

The decision-making process is formally defined by the tuple M=⟨S,A,P,R,γ⟩. The framework operates on two timescales. Monitoring is continuous; as the patient plays, Gesture Therapy streams kinematic data and the drift detector evaluates inference similarity over sliding windows in real time. Planning, by contrast, is episodic. The MDP is invoked at session boundaries, once a session has concluded and its aggregated kinematic, scoring, and self-report data are available, so that the full state s=⟨g,k,u,d,τ⟩ is observed before an action is chosen. The selected action then configures the following session. Decisions are therefore separated by one complete session, which in the present protocol corresponds to the day-on/day-off interval between scheduled sessions.

- **State Representation (S):** Each state encapsulates the patient’s current clinical and gaming condition as a vector s=⟨g,k,u,d,τ⟩ (Table 2):
  - g: Current game type.
  - k∈{Low,Medium,High}: Kinematic performance.
  - u∈{Low,Medium,High}: Task success rate, reflecting scores and mistakes (Low, Medium, High).
  - d∈{Low,Medium,High}: Game difficulty level (Low, Medium, High).
  - τ∈{↓,→,↑}: Performance trend derived from recent motion data (Improving ↑, Stable →, or Worsening ↓). For instance, τ is computed by comparing variables from the previous session to the data batch that triggered the drift.
  The components of the state vector are of two distinct kinds. The game type g and difficulty level d are control variables; they are configured by the system and are therefore known at the moment an action is selected. Kinematic performance k, task success u, and trend τ are outcome variables; they describe how the patient actually performed and are measured once a session has been completed.
- **Action Space (A):** The system selects from four targeted interventions, each of which also sets the difficulty of the next configuration. MaintainGame keeps the current game, with its level adjusted automatically following Sansores (16). SwitchWithinType moves to another game of the same category to maintain engagement. ProgressToNextType moves to the entry game, the lowest-difficulty game of the next category, to increase task complexity. RegressToPreviousType moves to the highest-difficulty game of the previous category to decrease task complexity. The signed change $\Delta d=d^{\prime }-d$ produced by each action drives the stochastic evolution of the outcome variables.
- **Transition Dynamics (P):** The incoming game $g^{\prime }$ is chosen deterministically according to the selected action a, and performance then evolves probabilistically as a function of Δd. The probability of each level for both task success and kinematic performance, sampled independently, is given in Table 3. A larger positive Δd raises the probability of a low outcome and a negative Δd favours a high outcome. The trend is then derived from the change in task success relative to the previous state, taking the value improving, stable, or worsening as success rises, holds, or falls.
- **Reward Function (R):** Built upon clinician feedback and validation, the reward function quantifies the clinical importance of various patient outcomes and establishes a hierarchy of therapeutic priorities. An improving kinematic trend receives the highest positive reinforcement (+50), a high task success rate keeps the patient motivated (+20), with an additional bonus when this is achieved at moderate difficulty (+10). The most severe penalty discourages task overload, where success is low while difficulty is high (−150). A worsening trend draws a heavy penalty (−75), and stagnation with low success draws a mild penalty (−20) to prompt intervention rather than allow a plateau (Equation 1):
  $$R(s^{\prime })=\left\{ \begin{array}{cc} +50, & \text{if }\tau^{\prime }=↑\\ +20, & \text{if }u^{\prime }=High\\ +10, & \text{if }u^{\prime }=High\wedge d^{\prime }=Medium\\ -75, & \text{if }\tau^{\prime }=↓\\ -150, & \text{if }u^{\prime }=Low\wedge d^{\prime }=High\\ -20, & \text{if }\tau^{\prime }=\to \wedge u^{\prime }\neq High\\ 0, & \text{otherwise} \end{array}\right.$$ (1)

**Table 2:** Variables used for MDP state representation.

Variable	Type	Description
Game Type (g)	Control	The current virtual rehabilitation game being played (e.g., Kitchen, Pond).
Difficulty (d)	Control	Current difficulty level of the game setting (Low, Medium, High).
Kinematic Perf. (k)	Outcome	Discretized overall kinematic efficiency (Low, Medium, High).
Task Success (u)	Outcome	Success rate reflecting scores and task mistakes (Low, Medium, High).
Trend (τ)	Outcome	Performance evolution from recent motion data:
		(↑ Improving, → Stable, ↓ Worsening).

**Table 3:** Probability of each performance level as a function of the difficulty change Δd (used by the training simulator for both task success and kinematic performance).

Δd	P(Low)	P(Medium)	P(High)
≥+2	0.7	0.2	0.1
+1	0.5	0.4	0.1
≤−1	0.1	0.4	0.5
0	0.2	0.6	0.2

### 3.2 Q-learning and policy deployment

The policy π(a|s) defines the agent’s decision schema, that describes the probability of selecting each possible action a∈A given the current state s∈S. Within the therapy planning framework, the policy encodes how the system decides whether to maintain, switch, progress, or regress the current game type based on the patient’s observed performance. This is achieved through interaction, in two distinct phases: a simulated Training Phase and a deterministic Deployment Phase.

#### 3.2.1 Training phase

To solve the MDP and derive the optimal therapeutic policy prior to real-world deployment, a simulated environment was developed to replicate the sequential therapeutic trajectories a patient might take. During the training phase, the agent interacts with this simulator over various episodes. In each iterative step of an episode, the agent executes the following cycle.

1. Observes the current simulated patient state $s_{t}$.
2. Selects a therapeutic action $a_{t}$ (for example, adjusting game difficulty).
3. Transitions to a new state $s_{t+1}$ according to the distributions in Table 2, tied to the difficulty adjustment.
4. Receives the corresponding reward $R(s_{t+1})$ and updates its internal value estimates.

Each episode was initialized in the Kitchen game with a stagnated trend, while kinematic performance, task success, and difficulty were each drawn uniformly at random from Low, Medium, High. The agent was trained over 20,000 episodes of 30 steps each. The proposed Q-learning approach allows the agent to build its policy entirely through interaction and observed outcomes, establishing a flexible architecture capable of handling the complex, non-stationary nature of real-world therapy data.

#### 3.2.2 Learning algorithm and clinical interpretation

During the simulated training, the agent updates its estimated action-value function, Q(s,a), using the Bellman equation (Equation 2):

$$Q(s,a)\leftarrow Q(s,a)+\alpha \left[R(s^{\prime })+\gamma \max_{a^{\prime }}Q(s^{\prime },a^{\prime })-Q(s,a)\right]$$ (2)

Here, α represents the learning rate, and γ is the discount factor, weighting the importance of long-term therapeutic gains over immediate successes. To ensure the agent discovers effective strategies without settling into sub-optimal routines, it employs an ε-greedy exploration policy, with ε initially high to encourage broad exploration and lowered exponentially as training progresses.

The hyperparameters were set as follows. The learning rate began α=0.1 and decayed as α=0.1/(1+ep/2,000), the discount factor was γ=0.9, and the exploration rate decayed exponentially from ε=1.0 to a floor of ε=0.01 with decay factor 0.9995. Bootstrapping was cut off on the final step of each episode. The agent was trained over 20,000 episodes of 30 steps from a fixed random seed.

In a clinical context, this learning mechanism corresponds directly to an iterative process of clinical reasoning. Early in the training, the agent explores the space boundaries to see how a patient responds to newly introduced stresses, and evaluates how each decision affects the patient’s future performance trajectory. Actions leading to sustained improvement are positively reinforced, while those associated with worsening fatigue or failure are penalized. Over time, as ε decays, the system learns these patterns, shifting from exploring unknown actions to exploiting the most successful, empirically grounded regimens.

#### 3.2.3 Deployment phase

Once the Q-values converge, the training phase concludes and the optimal policy ($\pi^{*}$) is established. For deployment with real patients during live sessions, ε is set to zero, transitioning the system to a purely greedy, deterministic policy (Equation 3):

$$\pi^{*}(s)=\arg\;\max_{a\in A}Q(s,a)$$ (3)

During this phase the framework operates as an active clinical decision-support tool. At the end of a session, whenever concept drift triggers an evaluation, the patient’s kinematic metrics, scores, and self-reported feedback are translated into the state representation s. The system then queries the optimized policy to select the action yielding the highest cumulative therapeutic reward learned during training. This two-step architecture, combining iterative hypothesis testing in simulation with deterministic execution at deployment, ensures that recommendations are aligned with safe, effective therapeutic reasoning.

## 4 Results and preliminary evaluation

### 4.1 Experimental design

To evaluate the proposed MDP framework, a longitudinal observational study was conducted over a four-week period. The experimental protocol was structured to ensure consistent data collection while mirroring a realistic rehabilitation schedule:

- **Study Duration & Schedule:** The study spanned 4 weeks using a day-on/day-off schedule, resulting in a total of 12 distinct data-collection sessions per participant.
- **Clinical Progression:** The intervention followed a progressive difficulty path aligned with the clinical game hierarchy. During Week 1, participants engaged with simpler compound movements (e.g., Kitchen, Grill, Library). By Week 4, the complexity scaled up to demanding, pressure-modulated tasks (e.g., Pond, Archery).
- **Session Structure:** Each session consisted of two 1.5-min rounds per scheduled game, yielding three minutes of active motor execution per task. To mitigate physical fatigue, a one-minute rest period was enforced between different games. For tasks introduced for the first time, a one-minute demonstration was provided.
- **Data Collection:** The Gesture Therapy system continuously recorded spatial coordinates and pressure logs. Post-session, participants completed a structured questionnaire to capture self-perceived metrics regarding task difficulty, personal performance, and overall expertise.

### 4.2 Participant characteristics

The study cohort consisted of three adult participants. They were specifically selected based on the following criteria to ensure the integrity of the collected kinematic data:

- **Demographics:** The cohort included two males and one female, with an average age of 52.3 years (individual ages: 46, 55, and 56). Two participants were right-handed, and one was left-handed.
- **Health Status (Unbiased Baseline):** Healthy individuals were selected for this phase of the study to establish an unbiased baseline of behavioral learning curves and system interaction. This strategy allowed the framework to accurately map the natural trajectory of skill acquisition prior to the added complexities of deploying the system with stroke survivors.
- **Digital Proficiency:** To ensure the kinematic data accurately reflected true motor adaptation rather than pre-existing digital dexterity, individuals with minimal prior gaming experience were recruited. One participant reported having “None,” while the other two reported “Low” gaming experience.

Participants specific characteristics are shown in Table 4.

**Table 4:** Participants profile characteristics.

Characteristic/Participant	LCV (P1)	ACV (P2)	TCV(P3)
Gender	Female	Male	Male
Age	56	55	46
Hand dominance	Left handed	Right handed	Right handed
Educational level	High school	High school	Technical career (Graphic Design)
Occupation	Housewife	Automotive industry employee	Graphic designer
Gaming experience	None	Low	Low

### 4.3 Semi-synthetic data generation

Semi-synthetic data were generated from the Bayesian network structure and conditional probability tables learned from the real-world data, so that the generated streams reproduced the distribution patterns of the real sessions under controlled conditions. For each participant and game, the number of generated samples matched the number of real samples in the corresponding session, preserving comparable conditions. Samples were produced by logic sampling, processing nodes in topological order so that parents are assigned before their children, with each node’s state drawn from its conditional distribution using a uniform random draw.

Drift was induced by dividing each generated stream into segments, with change points placed at random, and by perturbing the conditional probabilities between segments. Each probability was shifted by an amount drawn uniformly (Equation 4),

$$P^{\prime }(X_{i}=1)=P(X_{i}=1)+\Delta p,\;\Delta p∼U(-C_{p},\;C_{p}),$$ (4)

with the resulting value clamped to the interval [0,1]. The magnitude parameter $C_{p}$ controls how much the distributions vary between consecutive segments, with small values producing gradual change and large values producing abrupt change.

Two settings were generated. In the parametric setting a single structure was learned from all of a game’s sessions, and the conditional probability tables were then estimated separately for each session, so that drift appears as parameter change across sessions. In the structural setting an independent structure was learned for each session, so that drift appears as a change in the dependencies among variables. Each generated dataset retains the segment labels, the structures, and the conditional probability tables used, providing a ground truth for evaluating detection and adaptation.

### 4.4 System stability and drift assessment

A fundamental requirement for the adaptive planner is a reliable signal. On semi-synthetic data generated from the real-world networks, where ground truth is available, the framework maintained an average CPT similarity of 95.55% with a narrow standard deviation of 2.01% under parametric drift. Under structural drift it recovered structures with an average Structural Hamming Distance of about 0.20. These controlled results confirm that the detector adapts reliably to both parametric and structural change (12). On the fully real-world data, where no ground truth exists, the average inference similarity across all games was 63.68% with a narrow range of only 3.01%, indicating consistent performance despite the variability of the tasks.

The narrow similarity range across games indicates that the detector reliably captures the underlying distributions despite the intrinsic variability of human motor execution, localizing genuine shifts in performance rather than reacting to arbitrary noise. Because the real-world data has no ground-truth structure, the parametric-vs.-structural distinction is demonstrated quantitatively on the semi-synthetic data, while the real-world setting reports the parametric adaptation results.

### 4.5 Policy convergence and baseline comparison

The greedy-policy change-rate fell monotonically across training, converging to a mean of 0.014 [0.011 std, 95% CI (0.006, 0.022)] across ten seeds, confirming the policy converges before deployment. To assess whether adaptation helps, the learned policy was compared with non-adaptive baselines by mean cumulative reward over 3,000 simulated episodes of 30 steps:

The learned policy outperforms every non-adaptive baseline by a wide and consistent margin, and the fixed escalating schedule, which mirrors the rigid data-collection protocol, performs worst (Table 5).

**Table 5:** Mean cumulative reward of the learned policy vs. non-adaptive baselines (n=10 seeds).

Policy	Mean reward	SD	95% CI
Learned policy	+3.9	4.1	[0.9,6.8]
Always-maintain	−329.6	1.7	[−330.9,−328.4]
Random	−488.0	5.9	[−492.2,−483.8]
Fixed escalating schedule	−1,214.2	3.7	[−1,216.9,−1,211.5]

Across the 36 real sessions the action distribution was stable across seeds and leaned toward progression: ProgressToNextType 15.0±1.7 (95% CI 13.8 to 16.2), RegressToPreviousType 10.3±1.3 (9.4 to 11.2), MaintainGame 5.7±0.8 (5.1 to 6.3), and SwitchWithinType 5.0±1.2 (4.2 to 5.8), across ten seeds.

To assess the policy’s dependence on the assumed dynamics, each entry of the transition table was perturbed by a uniform amount, the table was renormalized, and the policy was re-derived across seeds. Perturbations of ±0.05 to ±0.15 changed the recommended action in 12 to 20 percent of states (12.3 percent at 0.05, 19.2 percent at 0.10, and 19.9 percent at 0.15), compared with an 18 percent disagreement between independently trained policies under different random seeds with no perturbation. The progression-leaning structure of the policy was preserved throughout.

#### 4.5.1 State-variable redundancy (k vs. τ)

To test whether kinematic performance k and the trend carry independent value, each was varied alone while the other state components were held fixed. Varying only k changed the recommendation in 17 out of 63 contexts (27%), and varying only the trend changed it in 44 out of 63 contexts (70%). Across the 36 real session states the correlation between k and the trend was −0.18. Both variables therefore carry independent discriminative value and are only weakly correlated, so they are not redundant, although k is the less influential of the two.

#### 4.5.2 Longitudinal performance trends

**Figure 5:**
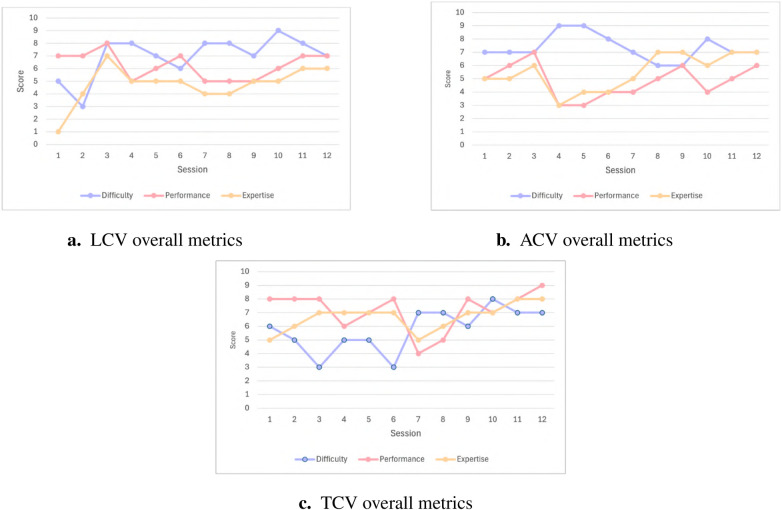
Self-reported difficulty, performance, and expertise across the twelve-session program for the three participants. Each panel shows one participant, with all three measures rated on a 1 to 10 scale after each session. Expertise rises across the program for all three participants, while performance dips when a new game category is introduced and recovers over the following sessions. **(a)** LCV overall metrics. Self-reported difficulty, performance, and expertise for participant P1 (LCV) across the twelve sessions. **(b)** ACV overall metrics. The same three measures for participant P2 (ACV). **(c)** TCV overall metrics. The same three measures for participant P3 (TCV).

Self-reported measures collected after each session provide a longitudinal view of the three participants across the twelve-session program (Figure 5). Self-rated expertise increased over the program for all three participants, from 1 to 6 for P1, 5 to 7 for P2, and 5 to 8 for P3, consistent with steady skill acquisition. Self-rated performance followed a recurring pattern, dropping when a new game category was introduced and recovering over the following sessions. These transition points are where the planner must decide whether to advance, switch, or hold, so the observed trajectories align with the decisions the policy is designed to make.

**Figure 6:**
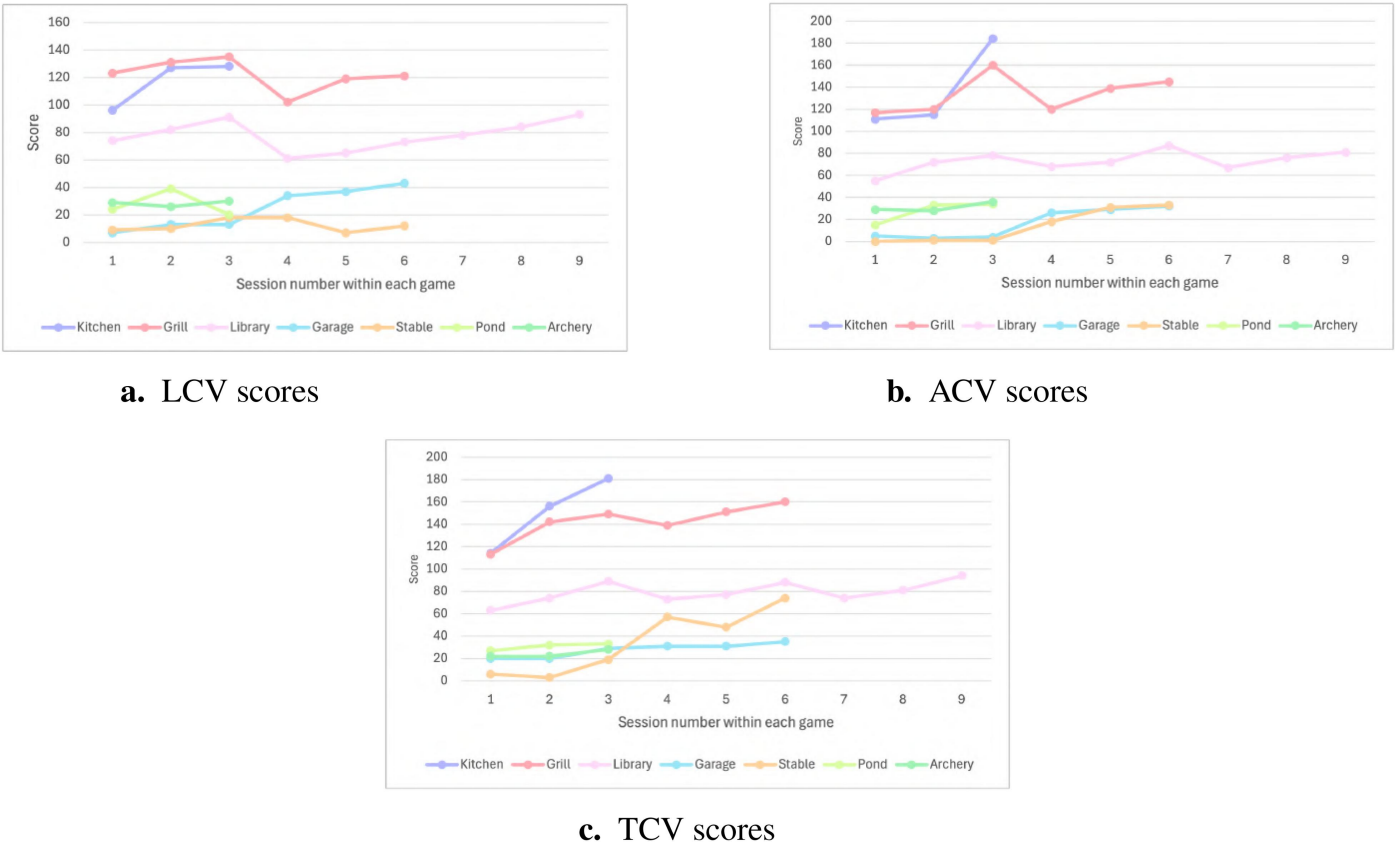
Objective in-game point scores per game across the sessions in which each game was played, for the three participants. Scores increased within most games for all three participants. **(a)** LCV scores. Objective in-game point scores per game for participant P1 (LCV), plotted against the session number within each game. **(b)** ACV scores. Per-game point scores for participant P2 (ACV). **(c)** TCV scores. Per-game point scores for participant P3 (TCV).

Objective in-game point scores corroborate this trend (Figure 6). Because point accumulation differs by game, scores are reported per game and are not comparable across games. Within each game, point scores generally increased over the sessions in which the game was played, for all three participants, with the clearest gains in Kitchen, Garage, and Stable. Point totals increased from the first to the last session in 19 out of 21 game trajectories (5 of 7 for P1, 7 of 7 for P2 and P3).

### 4.6 Adaptive policy recommendations

When concept drift triggered the MDP, the system processed the data streams and self-reported metrics to output a recommended action. The learned policy successfully demonstrated a balance between challenging the participant and providing stability, effectively mirroring clinical reasoning.

Below are representative examples of the system’s decision-making logic in practice:

- **Rewarding Improvement:** A participant interacting with a compound-movement game (Grill) exhibited high kinematic performance, high task success, and an improving data trend on a low-difficulty setting. The MDP accurately identified this as mastery of the current state and recommended ProgressToNextType, safely advancing the participant to a more complex motor category.
- **Mitigating Overload:** A participant engaged in a complex, pressure-modulated game (Pond) demonstrated low performance, low task success, and a worsening trend on a high-difficulty setting. The system recommended RegressToPreviousType to reduce task complexity and ensure a complete learning process before advancing further, potentially recognizing the indicators of fatigue or task overload.
- **Sustaining Engagement:** A participant showed stable trends but mixed performance, such as low kinematic efficiency but medium task success in Archery, the system recommended SwitchWithinType. By offering a lateral shift, the system potentially prevents frustration and maintains patient engagement.

### 4.7 Expert clinical review

The framework was reviewed by a single expert physiotherapist specialized in post-stroke and virtual rehabilitation, with experience using Gesture Therapy in rehabilitation sessions with stroke patients. The review had two parts. The expert first examined the overall design of the system, including the state representation, the action set, the transition and reward functions, and the resulting policy. The expert then evaluated the system’s per-session recommendations over two matched sets of cases, 36 real-world sessions from the three participants and 36 semi-synthetic sessions, for a total of 72 cases.

For the real-world cases, the expert was given the participant’s profile, the controlled session settings (the game and its difficulty level), and the video recording of the session, and noted the clinically relevant observations identified, such as signs of progression, improvement, or fatigue. For the semi-synthetic cases, where performance values were generated rather than recorded, the expert was instead shown the generated state and trend values describing each case and judged it on that basis. In both settings the expert selected one of the four available actions, MaintainGame, SwitchWithinType, ProgressToNextType, or RegressToPreviousType, as their own recommendation before the system’s recommendation was revealed, and was then asked whether they agreed.

The expert therefore judged the final recommendations rather than the inferred network states, and because the expert’s choice was recorded before the system’s output was shown, the evaluation was blinded to the system’s recommendation. As a single expert conducted the review, inter-rater agreement was not applicable. Agreement between the expert and the system was reached in 87.5% of cases, accounting for 63 out of the 72 total reviewed cases.

Because the evaluation relied on a single expert physiotherapist, the reported agreement reflects one clinician’s judgment and may vary across practitioners. A multi-expert evaluation, which would also allow inter-rater agreement to be quantified, remains future work. The physiotherapist who helped elicit the reward priorities also participated in evaluating the recommendations. This overlap is a potential source of evaluation bias, since the policy was assessed in part against the same clinical preferences that shaped its reward function, and the agreement figure should be read with that in mind.

By systematically rewarding improvement and heavily penalizing deterioration at high difficulty levels, the system produced session trajectories that experts judged consistent with rehabilitation reasoning. Whether these trajectories enhance motor recovery in clinical populations remains to be tested.

## 5 Discussion

Static rehabilitation protocols often struggle to keep pace with the dynamic, non-linear nature of motor recovery. To address this, we propose a framework that integrates concept drift detection within Bayesian Networks to drive a Markov Decision Process for adaptive therapy planning.

By monitoring data streams drawn from variables such as kinematic behaviour and task success, the system can identify both parametric and structural shifts in a user’s motor control. These detected drifts serve as signal for the MDP, which recommends a corresponding therapeutic adjustment. As a proof of concept, the framework was evaluated using data from healthy participants and assessed through expert clinical feedback. In this preliminary evaluation, the generated policies balanced engagement with progression, rewarding improvement while penalizing the risks of task overload and frustration, and the experts judged the recommendations consistent with established rehabilitation reasoning.

This integration of probabilistic tracking and sequential decision-making offers a promising basis for personalized, data-driven virtual rehabilitation, in which therapeutic adjustments can evolve alongside the user. The current results demonstrate technical feasibility rather than clinical effectiveness, and validation in stroke survivors remains the essential next step before claims of rehabilitation effectiveness can be substantiated.

Real stroke patients may respond differently from the simulated model. Improvement may be slower, fatigue may set in earlier, and the same increase in difficulty may carry a higher probability of decline. If the simulator understates these effects, the learned policy could advance patients too aggressively. Three features of the framework limit the consequences of such miscalibration. First, the action space is constrained by the game hierarchy, so any single decision moves the patient at most one step along the difficulty progression rather than to an arbitrary level. Second, the reward function is deliberately asymmetric, with the penalty for task overload far exceeding any positive reward, which biases the policy toward conservative adjustments in precisely the regime where miscalibration is most harmful. Third, the system is intended as a decision-support tool rather than an autonomous controller. In the early in-clinic sessions the therapist accepts or overrides each recommendation, and this feedback refines the policy, so that later, in home-based sessions, the system can be used more autonomously without approval of every individual action.

The reward magnitudes were elicited with the collaborating physiotherapist by ranking the clinical priority of each outcome (improvement, sustained success, deterioration, and overload at high difficulty), and assigning values that preserved that ordering, with the overload penalty set far larger than any positive reward to reflect its clinical seriousness. A sensitivity sweep of the reward weights showed the policy is robust to the magnitude of the overload penalty, and run-to-run variation exceeds the effect of individual weight changes.

The policy is about as sensitive to moderate changes in the transition probabilities as it is to the stochasticity of training, so the recommendations are robust to the precise calibration of the simulator dynamics, although the transition model remains an assumption that should be revisited with stroke-patient response data.

### 5.1 Limitations

While the proposed framework successfully demonstrates the viability of a drift-aware decision-making system in motor rehabilitation, several limitations must be acknowledged.

First, this study constitutes a proof-of-concept evaluation. The cohort comprised three healthy adults rather than stroke survivors, selected to establish an unbiased baseline of learning curves and system interaction. While this isolates the natural trajectory of skill acquisition, it does not capture impaired or compensatory motor behaviour, and the results therefore cannot speak to clinical efficacy in neurological populations. The findings should be read as evidence of technical and conceptual feasibility rather than rehabilitation effectiveness.

Additionally, the real-world validation was conducted using a fixed game schedule required by the data-collection protocol. This rigid structure occasionally forced artificial transitions between games, introducing bias that did not necessarily reflect the MDP’s reasoning or the participant’s actual state.

The current MDP formulation assumes that transition probabilities and reward dynamics remain stationary across sessions. In reality, patient performance and recovery trajectories may exhibit non-stationary behaviour as motor function returns or as long-term fatigue sets in. The transition model used during training rests on assumptions about how performance responds to changes in difficulty, informed by clinician feedback and by the behaviour of the healthy cohort rather than by the response curves of stroke patients. The learned policy is therefore only as well calibrated as those assumptions.

More fundamentally, several core components of the framework are specified through clinical expertise rather than learned from data. The reward function encodes expert judgment of therapeutic priorities rather than being derived from measured patient outcomes, and the game hierarchy, the action space, and the transition assumptions are likewise grounded in clinical reasoning and in the behaviour of the healthy cohort. The learned policy therefore reflects these expert assumptions and preferences, and clinicians who assign different reward priorities or define different therapeutic progressions could obtain different policies and recommendations. The framework should accordingly be understood as an expert-guided decision-support system rather than a fully automated, data-driven one. Grounding the reward function more directly in empirical patient outcomes is an important direction for improving its autonomy and generalizability.

Finally, the framework currently relies on the discretization of continuous kinematic variables into segments to function within the discrete Bayesian Network structure, which may result in a loss of granular continuous data.

### 5.2 Future work

Future research will prioritize extending the experimental validation to real clinical populations, in open-ended therapeutic schemes. Removing the constraints of a rigid experimental schedule will allow the MDP to guide the Gesture Therapy platform entirely autonomously, providing deeper insights into its practical clinical deployment.

Additionally, we aim to explore non-stationary or continuous learning models for the MDP. By allowing the agent to capture more gradual, continuous adaptations and integrate detailed feedback, the system will be able to better mirror the highly individualized nature of human neurological recovery and expert empirical knowledge.

## Data Availability

The raw data supporting the conclusions of this article will be made available by the authors, without undue reservation.
